# Sigmund Exner’s (1887) Einige Beobachtungen über Bewegungsnachbilder (Some Observations on Movement Aftereffects): An Illustrated Translation With Commentary

**DOI:** 10.1177/2041669515593044

**Published:** 2015-09-30

**Authors:** Frans A. J. Verstraten, Diederick C. Niehorster, Wim A. van de Grind, Nicholas J. Wade

**Affiliations:** School of Psychology, The University of Sydney, Australia; Helmholtz Institute, Utrecht University, The Netherlands; Department of Psychology, The University of Hong Kong, China; The Humanities Laboratory and Department of Psychology, Lund University, Sweden; Helmholtz Institute, Utrecht University, The Netherlands; Psychology, University of Dundee, UK

**Keywords:** aftereffect, adaptation, motion, rivalry, depth, color, interocular transfer, retinotopy

## Abstract

In his original contribution, Exner’s principal concern was a comparison between the properties of different aftereffects, and particularly to determine whether aftereffects of motion were similar to those of color and whether they could be encompassed within a unified physiological framework. Despite the fact that he was unable to answer his main question, there are some excellent—so far unknown—contributions in Exner’s paper. For example, he describes observations that can be related to binocular interaction, not only in motion aftereffects but also in rivalry. To the best of our knowledge, Exner provides the first description of binocular rivalry induced by differently moving patterns in each eye, for motion as well as for their aftereffects. Moreover, apart from several known, but beautifully addressed, phenomena he makes a clear distinction between motion in depth based on stimulus properties and motion in depth based on the interpretation of motion. That is, the experience of movement, as distinct from the perception of movement. The experience, unlike the perception, did not result in a motion aftereffect in depth.

## Introduction

One cannot understand the current status of a research field without having knowledge about its history. Understanding the past is not always easy, especially when the main scientific language changed in the course of history. That alone, however, does not justify translating scientific papers. The current article by Exner contains intriguing experiments and descriptions of binocular combination and interaction that make a full translation a worthwhile endeavor. Exner’s general goal was to discover the physiological basis for perceptual and cognitive phenomena. In the context of the motion aftereffect (MAE See Wade, 1994; Mather et al, 1998; Wade & Verstraten, 2005), it was to determine where it originates and how it compares to other aftereffects like those for luminance and color. His conclusion “that the question remains unanswered” may not be the best invitation to read the paper. However, the many interesting observations make this article a treat for the mind.

We have tried to follow the original text as closely as possible. The goal, however, was to convey Exner’s ideas. We adjusted the text when necessary for understanding his ideas. Exner’s article did not contain any illustrations. We have added the illustrations for reasons of clarity. Truth be told, it was not always clear what the exact viewing conditions were (see e.g., Figures 5 and 6), but we think we captured the essence.

## About Sigmund Exner (1846–1926)

**Figure 1.**
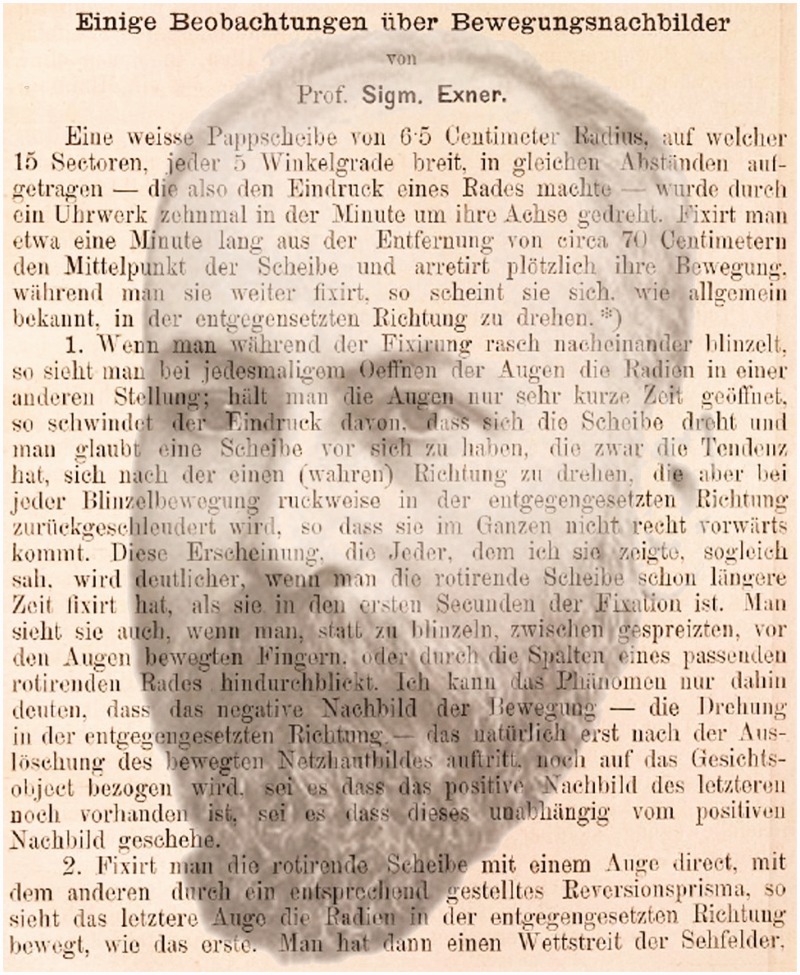
A portrait of Sigmund Exner combined with the first page of his 1887 article on the movement aftereffect (by Nicholas Wade).

Exner (see Figure 1) was born in Vienna, where he spent most of his life and where he died. After completing his medical studies at the University of Vienna, he became an assistant to Ernst Wilhelm von Brücke (1819–1892) and later worked with Hermann von Helmholtz (1821–1894) in Heidelberg. In 1891, he became the head of the Institute for Physiology of the University of Vienna, as successor to von Brücke. Exner was one of the teachers of the Gestalt psychologist Max Wertheimer, and he was undoubtedly one of the most important scientists of his time. He examined the apparent motion of brief flashes (Exner, 1875b) and introduced the term *reaction time* (Exner, 1873). Following his research on the facetted insect eye (Exner & Eckhard, 1891), he is regarded as one of the founding fathers of the field of comparative physiology, and he worked on a wide range of other topics, including cortical localization (Exner & Eckhard, 1881).

The current article is predominantly about the MAE, although Exner referred to them as motion afterimages. The MAE was not a novel phenomenon when Exner examined it, and he cited some of the earlier (mostly German) studies relating to aspects of it. The appearance of illusory motion on a static scene following observation of flowing water was described by Aristotle and Lucretius but came to prominence with Addams’ (1834) description and interpretation of apparent motion in stationary rocks following observation of a waterfall. The phenomenon later became known as the *Waterfall Illusion* probably because Thompson (1880) referred to it as the Waterfall Effect (see Verstraten, 1996; Wade & Verstraten, 1998). It can, however, be produced with many varieties of motion. The translation of Exner’s article follows.

## Translation of “Einige Beobachtungen über Bewegungsnachbilder”

Centralblatt für Physiologie 11. Juni 1887 № 6.

Some observations on movement aftereffects

By Prof. Sigmund Exner

**Figure 2.**
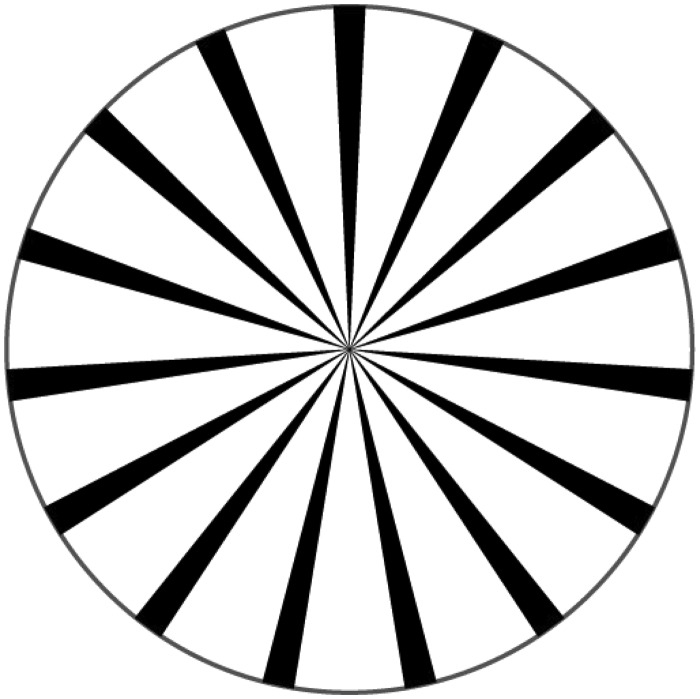
A representation of the sectored disk used by Exner.

A white cardboard disk with a radius of 6.5 cm, which contains 15 sectors, each spanning 5° of arc and placed at equal distances—so that they give the impression of a wheel—was rotated around its axis by a clockwork at a rate of 10 revolutions per minute. When one fixates the center of the disk for about a minute, from a distance of about 70 cm and suddenly stops its movement, while maintaining fixation, the disk, as is generally known, appears to rotate in the opposite direction (Figure 2).

**Comments:** The rotary MAE produced with radial patterns was examined by Wundt (1874) and Aitken (1878). Prior to that the most common stimulus for inducing MAEs was a rotating spiral (Plateau, 1849). Linear motion of gratings was suggested by Addams (1834) and effected by Oppel (1856) and Bowditch and Hall (1882).

*Noticeably,^1^1Note by the translaters: the additions in italics were originally published as footnotes in Exner's paper. with continued fixation, it seems to me that, while the disk is actually moving, some of the spokes disappear, while the movement of the others is recognized correctly. It even happened for short periods that I could only see 5 of the 15 spokes. However, these then appear very sharply defined. It also happens that only the inner half of a spoke is visible. Any movement of the eyes makes this illusion fade away.*

**Comments:** Partial and complete disappearances of stationary geometrical patterns were described by Troxler (1804), Brewster (1818), and Purkinje (1819/1823). Exner extends this to rotating patterns and draws attention to the importance of steady fixation. Also interesting is mentioning of the disappearance of some of the spokes. This could be related to, but seems to be clearly different from, two phenomena. One is known as motion-induced blindness (Bonneh, Cooperman, & Sagi, 2001) and the other time-locked fading (Kanai & Kamitami, 2003).

1. If one, while fixating, rapidly and successively blinks, one sees the spokes in a different location each time the eyes are opened; if the eyes are opened only for a very short time, the impression that the disk is rotating fades, and one has the impression of looking at a disk, that, although it has the tendency to move in the (veridical) direction, still seems to be pulled back in the opposite direction every time you blink, so that it appears as if the whole does not really move forward. This phenomenon, that everyone that I have shown it to, immediately saw, becomes clearer, when one has fixated the rotating disk for a longer time, as compared with the impression you get during the first seconds of fixation. One can also see this when, instead of blinking, outspread fingers are moved in front of the eyes, or if one looks through the gaps of a suitable rotating wheel.

**Comments:** The apparent reversal of direction after blinking could be the consequence of the eye returning to its normal orientation after optokinetic torsion. This explanation makes sense as Exner confirms that the effect becomes stronger in time. The others are probably related to the successive stimulations that are experienced with stroboscopic disks, which would have been widely known in Exner’s time (see Mannoni, 2000; Wade, 2004). Purkinje (1819/1823) employed the technique of waving fingers in front of the eye as well.

I can only explain this phenomenon by the negative afterimage of movement—the rotation in the opposite direction—which of course only occurs after the extinction or disappearance of the moving retinal image, is still attributed to the visual object, either because the positive afterimage of the latter is still present or because this happens independently of the positive afterimage.

**Comments:** Exner was concerned with relating aftereffects of motion to those in other domains, like color, since they all appear like the negative of what was perceived during adaptation. More specifically, he wishes to determine whether the MAE is retinal or due to higher processes located in the brain. Thus, in the last sentence, he makes clear that there is at least an afterimage of the stimulus that activated the retina, and he wants to find out whether the aftereffect of motion is attributed to the positive aftereffect of the wheel on the retina (the aftereffect shows up immediately so it must be attached to it, if it is retinal) or whether this aftereffect occurs irrespective of what remains on the retina, that is, when nothing is left in terms of afterimages on the retina.

2. If one fixates the rotating disk directly with one eye, and through a suitably positioned reversion prism with the other eye, then the latter eye will see the spokes move in the opposite direction compared with what the first eye sees. One then has a rivalry of the visual fields of the two eyes, while the spokes at different places of the disk move in opposite directions, and here and there they also seem to pass through each other; the whole gives a restless impression.

If one has fixated in this manner for a minute and the motion is stopped, no clear illusory movement is observed on the disk. One can certainly doubt whether the disk with all its spokes are seen at rest as it was for the case when the disk had not moved before, but one cannot doubt that the movement aftereffect, as compared with the cases in which one observes without a prism or with only one eye, is reduced to a minimum.

*Already several years earlier, Dvořák (1870) had noticed that the aftereffect does not show up, if two oppositely directed motions are presented on the same retina.*

When I observed the disk for a minute in the same manner, and closed one eye at the same time the disk was stopped, I experienced the negative aftereffect that belongs to the opened eye. By alternatingly opening and closing the two eyes, I could even make the illusory motion of the disk change its direction. However, the clarity of the movement aftereffect for each eye in this condition is much less than if both eyes had looked at motion of the disk in the same direction or if both the movement of the disk and its aftereffect were observed with only one eye.

**Comments:** These are some of the most intriguing observations. By reversing the rotation of the sectored disk in one eye (by means of a reversing prism) relative to the other, he provides probably the earliest statement regarding *binocular rivalry with moving patterns*. The first description of the phenomenon is usually attributed to Breese (1899), although he only moved the pattern (a grating) in one eye (see Wade & Ngo, 2013). Exner takes the phenomenon further and examines the aftereffect of rivaling motions: Opposite MAEs can be seen when a single eye observes a stationary pattern, but little or no MAE is visible when both eyes are open (as Dvořák 1870 had noted earlier. See Broerse et al., 1994 for a translation.). Moreover, the fact that the clarity is less when both eyes are stimulated by opposite motion directions points to interocular inhibition. Some of these results have been confirmed more recently, but without reference to Exner’s work (see Feresin, Wade, & Swanston, 2001; Moulden, 1980).

3. I observed a real battle between movement aftereffects in the following way: The drum of a Ludwig kymograph (purchased from Balzar) was spanned with lined paper. The lines were vertical, 1.5 mm thick, and each line had a distance of 5 mm to its neighbors. *This is the same device that my colleague Fleischl v. Marxow (Fleischl, 1883) used for his observations*. The drum makes a little more than 3 revolutions per minute.

**Figure 3.**
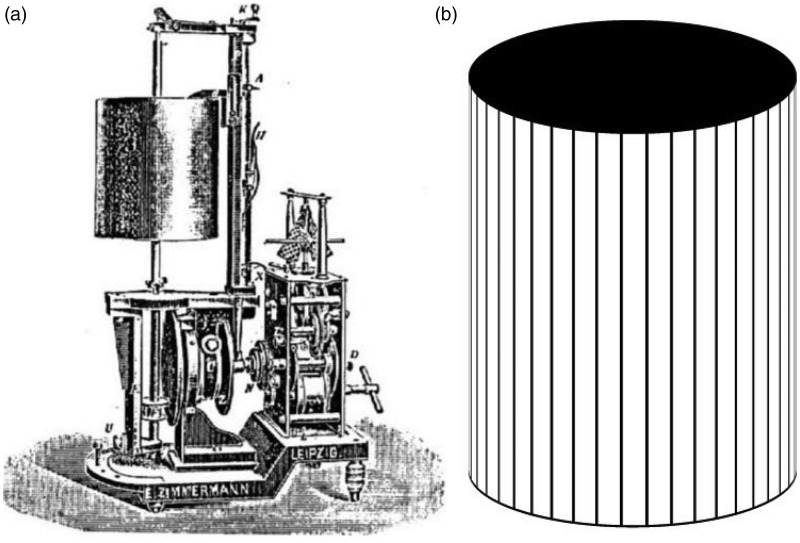
(a) An illustration of the Ludwig-Baltzar (in the original paper Exner wrote Balzar) kymograph (wave writer) named after the famous physiologist Carl Ludwig and the precision engineer Gerhard Baltzar. Ludwig designed the machine and Baltzar produced it. This diagram is taken from the famous Zimmermann (1903/1983) Scientific Instrument catalogue. (b). The drum was lined with paper as described by Exner.

If one looks from a distance of about 80 cm and suddenly stops the movement, a movement aftereffect is obviously experienced. If one now looks with one eye through a reversion prism, such that the lines physically proceeding from left to right are seen as moving from the bottom to the top, rivalry of the visual fields is again perceived if both images of the drum are made to superimpose as far as this is possible. If one now stops the drum, a peculiar undulation of the two line systems shows up, which one recognizes as the expression of rivalry between the two aftereffects. Also, individual groups of lines of one direction with their illusory motion become visible as well as groups of the other movement direction (Figure 3).

**Comments:** Binocular rivalry between orthogonal moving gratings is described initially; the perceptual outcome of rivalry is not as clear cut as with oppositely rotating sectored disks. Moreover, the aftereffects so generated rival with one another—again a novel observation. The “peculiar undulation” (German: eigenthümliches Wogen) probably reflects the fact that the combined aftereffects are in a diagonal direction with respect to those of adaptation. A clearer description of MAEs in resultant directions was given later by Borschke and Herscheles (1902). In the discussion of the results (see later), Exner is very clear. He first describes binocular fusion of color which either results in rivalry or binocular color mixing. However, he states that this does not happen for motion. Rivalry is experienced both during adaptation and testing. This is apparently not an all or none experience. Parts of the visual field, as we now know (Blake, 2001), show the aftereffect of one pattern, and other parts the other aftereffect. However, the two aftereffects do not integrate in the same spatial area.

**Figure 4.**
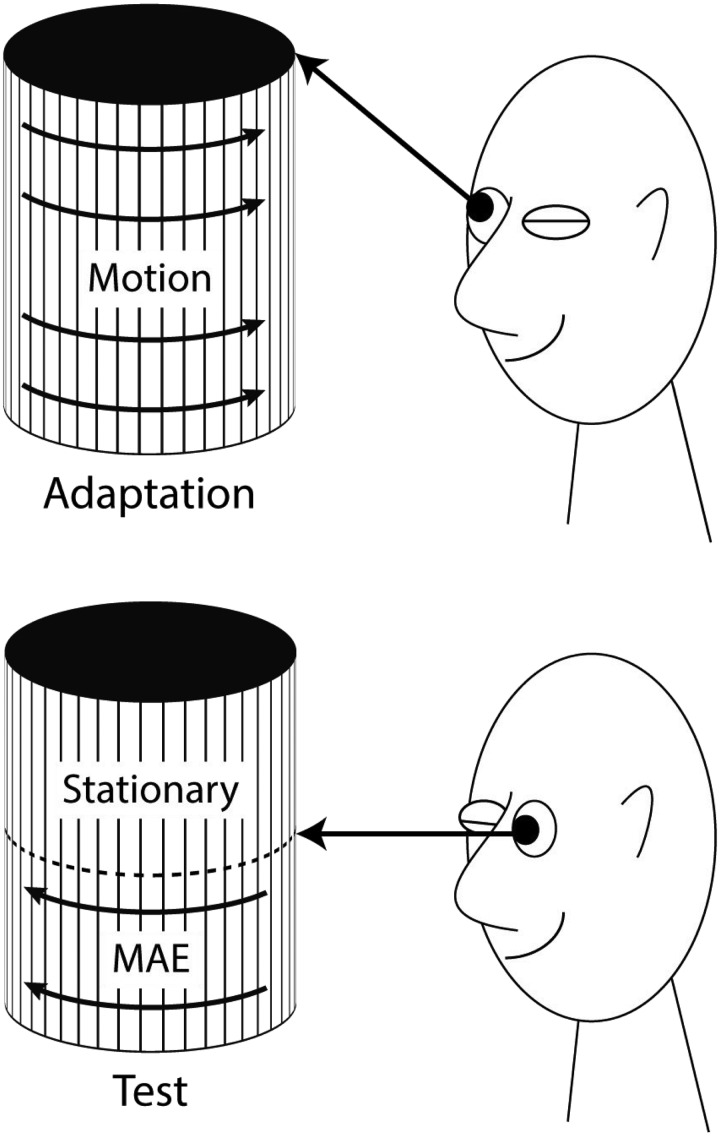
Exner’s Experiment 4 showing both interocular transfer and retinal localization of the MAE.

4. If one fixates the center of the moving drum (or a suitable marker placed just in front of it) with the right eye for a minute, subsequently stops the drum, and closes the right and opens the left eye at the same time, a negative movement aftereffect is experienced in the latter eye at the fixated lines. The experiment becomes really convincing if one has fixated a point on the upper border of the drum with the right eye and at then looks at the middle (at half height) of the stopped drum with the left eye. It then appears that only the lower parts of the lines undergo the aftereffect, the upper parts remain at rest (Figure 4).

*This transfer of the movement aftereffect to the unstimulated eye was already observed by Dvořák.* *Budde (1884)* *disagrees. Dvořák and also Bowditch (* *Bowditch & Hall, 1882* *) have pointed out that the afterimage is restricted to the area in the visual field that was stimulated.*

**Comments:** As Exner notes, Dvořák (1870) reported that an MAE generated with only one eye open can be seen with the other eye, although neither compared the MAEs quantitatively. Exner makes two observations here. First, he confirms the occurrence of interocular transfer when the center of the drum is fixated during adaptation and test. Second, he demonstrates the retinal localization of the MAE by fixating on the upper rim during adaptation and the center during test. This produces an apparent *shearing* above and below the point of test fixation.

5. As is generally known, one does not see rivalry of the visual fields easily when one eye is closed. As the result of frequently viewing through my right eye when using a microscope, I am accustomed to suppressing the input of my left eye, therefore—under otherwise unchanged circumstances—the impressions in my right eye enter consciousness more easily. This could well be the reason that, when I close my right eye, I experience rivalry of the visual fields relatively easily, where the mist of the dark visual field of the right eye or the red light that penetrates through the lid is in competition with the images of external objects that are projected on the left retina.

When I now look at the rotating drum with my left eye and close the right eye and cupping it with my hand, I perceive rivalry between the light dust that reaches the right eye and the moving lines. This light dust now has a lively streaming and waving movement, which is opposite to the movement of the lines. The experience is so striking for my right eye, that it forced itself upon me, when I made an observation for the first time in the outlined manner. When I do this for the left eye, however, I can at best see indications of the rivalry—if at all—and consequently of the streaming of the light dust.

*It is very easy to see the regular movement aftereffect in the* mist* of the dark visual field and to confirm the phenomenon emphasized by* *Zehfuss (1880), where, after the view was directed towards a window of a train wagon, at the time the eyes were closed a waving in the visual field of corresponding direction is perceived.*

**Comments:** By making reference to the research by Zehfuss, Exner was referring to the streaming or scintillation seen with closed eyes following observation of static or moving patterns. It had been described by Purkinje (1819/1823) and was amplified by Wohlgemuth (1911). Zehfuss noted the effect with sectored disks as well as viewing through a window of a moving train. The effects can be seen superimposed on the pattern during adaptation or following when observing a blank field with closed eyes (see Wade, 1977).

**Figure 5.**
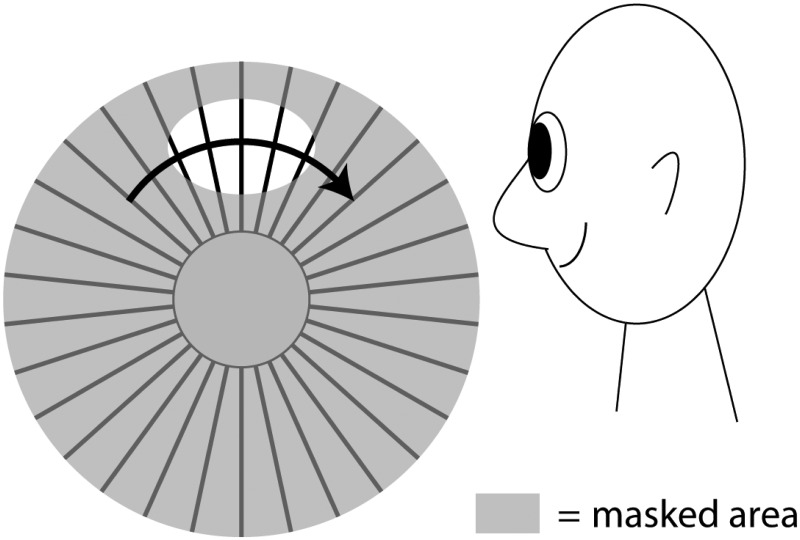
Representation of the experimental set-up for Experiment 6. Most of the wheel was covered as represented by the shaded area, where it should be noted that the extent of coverage cannot be ascertained from the text.

6. A star was constructed from 30 knitting needles in such a way that the end of each needle was at a distance of 21 cm from the center, and all needles, like the spokes of a wheel, were in the same plane and of course equidistant from each other (12°). The free part of the spokes was 15 cm long and oxidized by a vapour of nitric acid to avoid glare. The wheel was set in rotation, so that it made 8 revolutions per minute. I seated myself such that the plane with the spokes was lined up with my medial plane and fixated a mark placed perpendicular to the axis of rotation at about half the height of the free spokes in such a way that the spokes during rotation passed very close by the fixation mark. I got so close that the needles nearly touched my nose and screened off everything, except that part of the needles that where moving toward me: Also the ends of these needles were covered, as far as possible, to ensure that their up and downward movement would not misguide me. Under these conditions, it is possible to achieve an impression of motion in depth *(this impression does not persist to the full extent if one fixates for a longer time: it becomes less clear and goes astray. Yet, this does not mislead the observer regarding the rotation direction)* and to test whether this impression results in a negative afterimage (Figure 5).

When I fixated for 1 to 2 minutes and suddenly stopped the wheel, it undoubtedly appeared to make a backward movement. When I fixated in the same manner, or even for 3 minutes and subsequently looked at the sentences of a book, I could not find any trace of movement, in the sense of letters moving away from me. When I closed one eye, the letters showed the aftereffect corresponding to that of the retinal image motion of the needles, therefore they moved from left to right for the right eye, and from right to left for the left eye, this is because the needle images brushed the retinas in opposite directions. It also happens on monocular viewing of the printed page that rivalry of the visual fields shows up, at least in so far that parts of the perceived field moved in one direction, corresponding to the aftereffect of the closed eye, while other parts appeared to drift in the opposite direction.

**Comments:** With both eyes open, an MAE in depth is experienced, at least when Exner is looking at the now stationary stimulus. Thompson (1877) had reported MAEs in the third dimension, and Exner (1888) describes them more fully. This MAE in depth does not show up when Exner looks at sentences in a book after adaptation to the same stimulus. At least when viewed with both eyes. When viewed monocularly, the expected eye-based MAEs do show up. This seems to indicate that the MAE has built up but when there is no appropriate postadaption stimulus to which a MAE in depth can be attached, the aftereffects in both monocular channels cancel each other. This is an early form of the AND/OR gating discussion (see Van Kruysbergen & de Weert, 1994; Wolfe, 1986). Spokes rotating in the median plane results in differential motion stimulation for each eye, and it is this aspect that Exner is referring to when looking at text with the left and right eyes separately. But he also seems to indicate that there are parts of the visual field that show motion that is based on the motion of the closed eye. So both aftereffects seem to be visible and independent.

**Figure 6.**
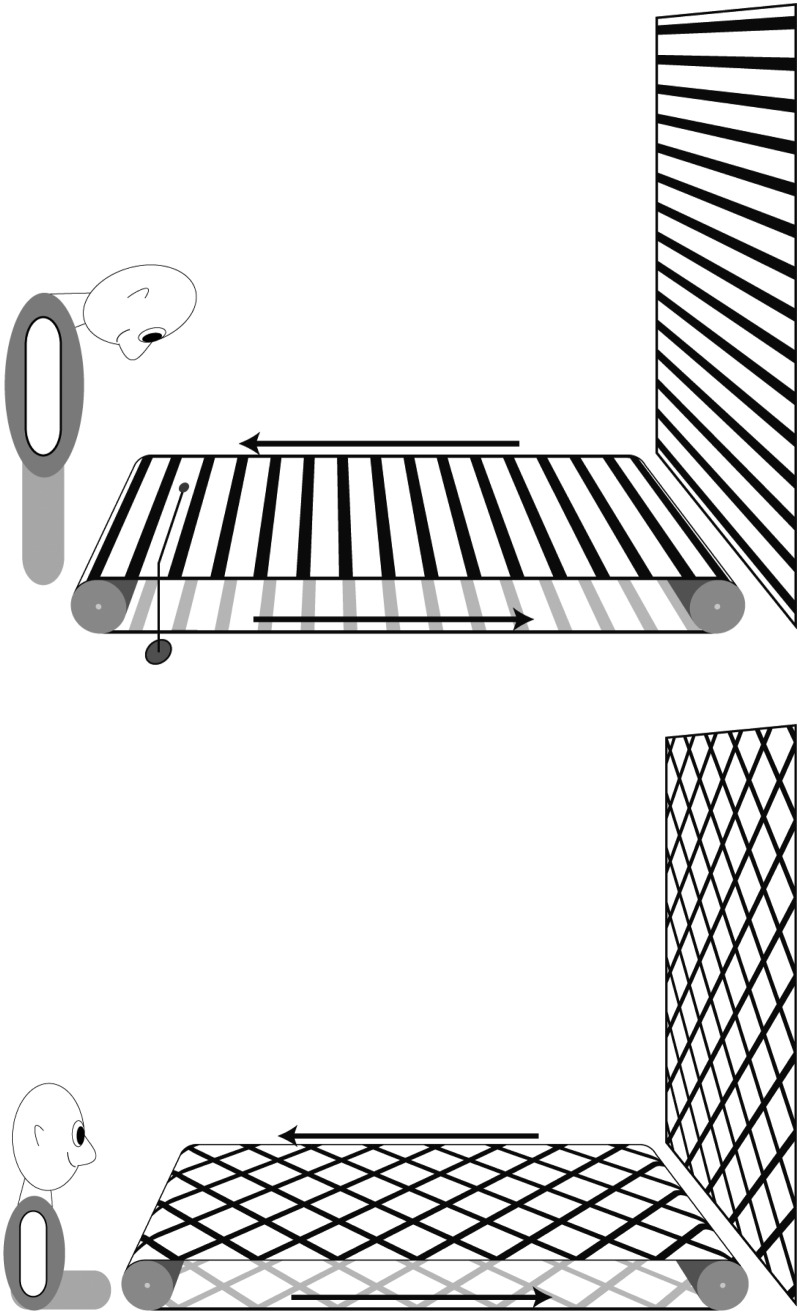
Assumed representation of the experimental set-up for Experiments 7 and 8. It is not particularly clear for both experiments what the test pattern looked like. But as we read it, it was a vertical stationary version of the adapting stimulus.

7. If one looks, while standing upright with head down perpendicularly to a horizontal plane on which lines are drawn from right to left that are moving toward the observer (a wide paper strip without endings ran over two horizontal rollers); fixate a mark placed close to the paper, and subsequently look, by lifting the head, at a vertical screen, an upwards directed MAE is experienced (Figure 6).

8. If one provides this belt with two new sets of lines tilted 45° relative to the previous lines so that they form squares and which diagonal lies in the direction of motion, and positions oneself such that the stimulus looks foreshortened and that the drawn stimulus moves toward you, one experiences a perfect impression of movement in depth. The movement aftereffect of this, projected on a vertical screen, is always directed from the bottom to the top, and I do not succeed in seeing even the slightest trace of movement away from me, and also no approach or distancing between the screen and a paper mark attached to a wire that hangs free in front of the screen.

**Comments:** The crossed diagonals resemble what is now often called *a plaid*. During adaptation the percept is one of movement in depth toward the observer. However, Exner cannot perceive the MAE to appear to move in depth. It remains unclear whether Exner used these vertical test patterns and was not just using the stationary version of the adapting stimulus configuration.

## Exner’s Discussion

The current experiments show that there are certain analogies between lightness- and color experience on the one hand and motion experience on the other hand, as well as their aftereffects, but that, from several perspectives, important differences also show up.

Movement experiences and their aftereffects are essentially restricted to those locations of the visual field that correspond to the parts of the retina that were directly stimulated, as is the case for color experiences and their aftereffects. (Whether also the directly adjacent areas are affected is hard to determine for movement aftereffects.) As is known, some people experience rivalry of the visual fields if identical retinal locations are stimulated by different colors; others may achieve binocular color mixing. With respect to the experience of motion, the latter will never occur, neither during the time the adapting stimulus is present nor while the aftereffect is experienced (Experiments 2 and 3), not even for those observers who are able to mix colors binocularly. The Right Honourable Hofrath von Brücke belongs to these, and kindly made the observations in my experimental set-up. However, I would like to emphasize, that for him also in binocular color mixing (a yellow and a blue circular disk on a gray background fused stereoscopically), the negative afterimage of each individual eye alone, when projected on a gray background, is the aftereffect of the veridical stimulus color, not of the mixing color. *Compare my treatises (Exner, 1875a, 1884, 1886)*. It is also the case for those, like me, who never totally get rid of rivalry, yet see a mutual dulling of the mixed colors, a really graceful experiment.

**Comments:** Although Exner does not perceive the color mixing as experienced by his colleague von Brücke, the dulling seems to leave space for interocular effects that can be regarded as mutual suppression.

The next difference that we can bring up between the perception of color and movement is that the experience of motion is immediately followed by the negative after effect (Experiment 1), whereas for color the negative afterimage is preceded by a positive afterimage. Also, the MAE obtained in one eye transfers to visual objects in the other eye (Experiment 4), for which there is no analogue in the field of color perception. Also, the decrease in movement aftereffect magnitude in one eye by presenting oppositely directed motion in the other eye (Experiment 2) belongs in this category. Not less the fact that corresponding locations in the nonstimulated eye reveal, in the mist of the dark visual field, a stimulation that corresponds to the negative aftereffect of the stimulated eye (Experiment 5).

Experiment 7, like all projections of movement aftereffects when changing our head and eye position or the inclination of the planes on which the aftereffect is projected, shows that we do not, as could be expected, have to do with an adjustment of our judgments for movements in certain directions, but with an adjustment of the physiological relations between neighboring retina locations or their central projections whose relations which, as I have highlighted some time ago (Exner, 1875b) give rise to the experience of movement, as distinct from the perception of movement. It is of importance, that, as shown by Experiment 6 and more specifically in Experiment 8, the experiences that underlie the perception of depth do not take part in the movement aftereffect: Even if our interpretation was changed to “approaching”, the subsequent experience of “receding” for objects at rest could not be achieved (for those cases, in which the illusory motion of the retinal images independently lead to a impression of depth are not considered here). So, it is not the physiological processes in the nervous system that underlie the judgments “movement upward,” “movement rightwards,” “movement towards me,” and so forth, that are changed by the preceding view of movement, but a physiological process, which happens either in the neuronal connections of anatomically defined retinal locations or in their more centrally located stages. Since it is meaningless in daily life, language has no name for this physiological process. It only occurs in combination with other excitations, and in this combination, it will result in judgements of the kind just mentioned.

**Comments:** It is difficult to interpret Exner’s failure to see an MAE in depth without a more precise appreciation of the viewing conditions. Similarly, the distinction between movement experiences and movement percepts is less than lucid. However, Exner considers the interpretation of the stimulus as moving in depth as a movement experience (as opposed to a movement percept), something that can only be seen with the actual stimulus and not during the MAE phase. It does work if the stimulus automatically gives the impression of depth (say with no “interference” of the will) like in Experiment 6 where Exner mentioned MAEs in depth. The situations in which MAEs in depth are seen use test stimuli that are similar to the adaptation stimuli; those in which no MAEs in depth are observed involve quite different test and adaptation stimuli.

Years ago, I have put forward the opinion, based on a series of experiments, that the afterimages of color and lightness experiences originate at the level of the retina. The current experiments were conducted to experience whether the same is true for movement aftereffects; the results, however, do not seem to be suitable to make any strong claims for one or the other notion, so that the question “Do the physiological processes that underlie the movement aftereffects occur in the retina or the brain?” for the time being must remain unanswered.

## Epilog

Exner’s principal concern was a comparison between the properties of different aftereffects, and particularly to determine whether MAEs were similar to those of color and lightness and can be encompassed within a unified physiological framework. He remained undecided upon this. Despite the seemingly sanguine conclusion, perhaps the greatest contribution of Exner’s observations can be related to binocular interaction, not only in MAEs but also in rivalry. To the best of our knowledge, Exner provides the first description of binocular rivalry induced by differently moving patterns in each eye. This applied to both rotating sectored disks and moving gratings by adopting the simple procedure of placing a reversing prism in front of one eye. The counter-rotating sectors would have been a better stimulus as they share a common center which assists bi-fixation. The orthogonally moving gratings might have had the tendency to induce optokinetic nystagmus in each eye.

Binocular rivalry between a static and moving patterns was described by Breese (1899). He presented a static green and black diagonal grating to one eye and a moving red and black grating of the opposite orientation to the other. The moving grating was fixed to a pendulum and was located behind a fixed square aperture. He reported that:

The moving lines of the red field were seen practically all the time. The stationary green field came and went with its usual regularity. The length of time it remained, however, was somewhat shorter than under normal conditions. While the green field was present the lines of the red field were still seen moving back and forth, seemingly *through* the lines of the green field. (pp. 30–31)

Exner referred to the counter-rotating sectors as sometimes seeming to pass through one another. This was not, however, the principal purpose of Exner’s experiment: He demonstrated that little or no MAE followed such adaptation when both eyes viewed the static test stimulus, but oppositely directed ones could be seen when each eye was opened in turn. The monocular aftereffects were less vivid than those following stimulation with a single direction of rotation. With orthogonally moving gratings the MAEs do engage in rivalry, and Exner’s description indicates that the rivalry can be piecemeal with parts of one grating seen together with parts of the other in neighbouring regions. Panum (1858) described and illustrated the piecemeal rivalry that can be seen with static orthogonal gratings.

**Figure 7.**
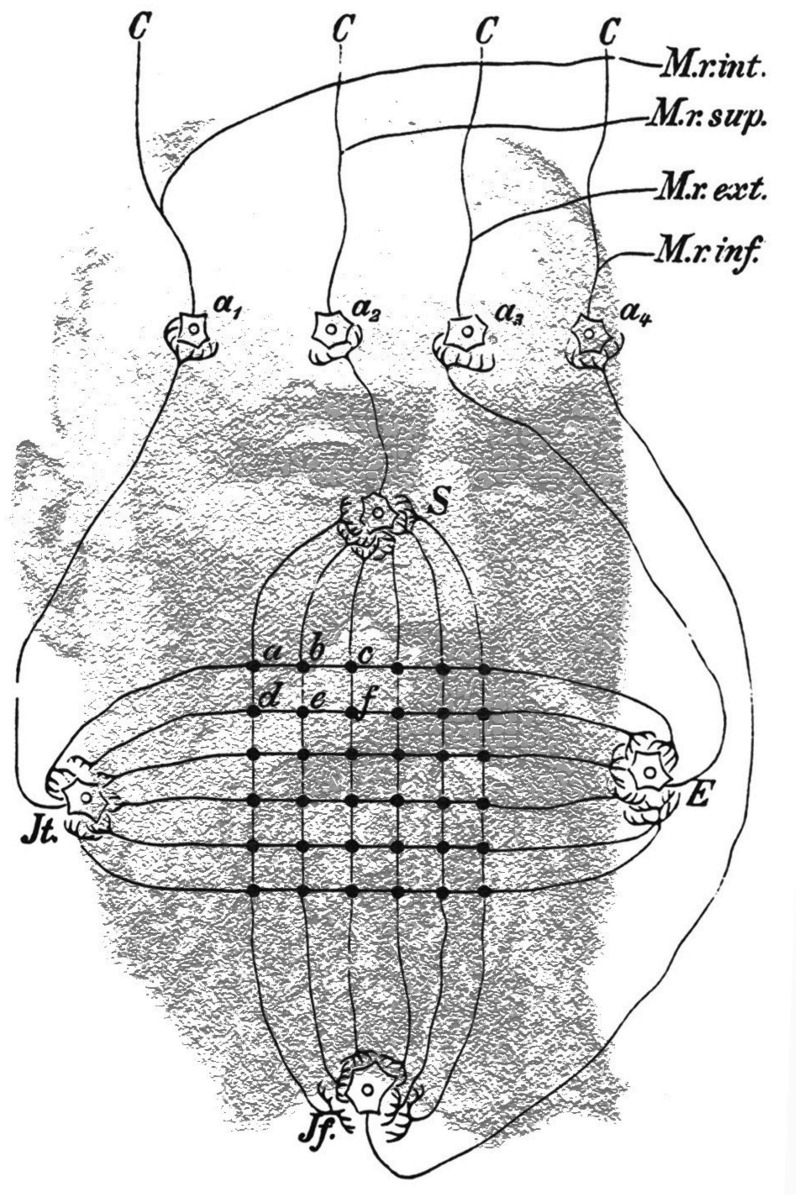
Sigmund Exner’s portrait is combined with his model of movement aftereffects taken from his *Entwurf* (1894, image by Nicholas Wade). See text for details.

Exner introduced his model of movement aftereffects in his *Entwurf* (1894) where he writes:

Schema for a centre of optical motion detection. *a-f* and the analogous points are the locations at which the fibres from the retinal elements enter the centre. The cells *S, E, Jt* and *Jf* represent centres to which each and every point of excitation arrive and where they can be summated. The time required by the excitations to reach there from any one of these points would be approximately proportional to the distance given in the diagram. *a_1_-a_4_* are centres which are closely related to, or perhaps identical with, the nuclei of the external eye muscles (only four are shown in the schema: M. rectus internus, superior, externus and inferior). *C* are fibres to the cortex, the organ of consciousness. (p. 193)

Exner elaborated on these observations in an article published in the next year and in his book on physiological interpretations of psychological phenomena (Exner 1888, 1894). It is in the latter that he presented his model of movement aftereffects; his portrait is combined with the model in Figure 7. Exner’s model was expressed in terms of influences on eye muscles, but it could be interpreted more generally. The symbols *a–f* denote units receiving input from retinal receptors, which will be stimulated in succession, dependent on the direction of motion. Prolonged stimulation in a given direction (e.g., *a–c*) will lead to the fatigue of the summation cell *E*, but not of *Jt*; this imbalance will be displayed when a stationary stimulus is subsequently viewed. Exner was also the source of inspiration for a similar, but more generally stated, analysis of motion presented by Stumpf (1911; see Todorovic, 1996) and MAEs (see Sutherland, 1961). Parts of this text were previously published in Wade and Verstraten (1998).
